# Post-traumatic growth following acquired brain injury: a systematic review and meta-analysis

**DOI:** 10.3389/fpsyg.2015.01162

**Published:** 2015-08-14

**Authors:** Jenny J. Grace, Elaine L. Kinsella, Orla T. Muldoon, Dónal G. Fortune

**Affiliations:** ^1^Acquired Brain Injury IrelandEnnis, Ireland; ^2^Department of Psychology, Centre for Social Issues Research, University of LimerickLimerick, Ireland

**Keywords:** brain injury, head injury, trauma, post-traumatic growth, rehabilitation outcomes

## Abstract

The idea that acquired brain injury (ABI) caused by stroke, hemorrhage, infection or traumatic insult to the brain can result in post-traumatic growth (PTG) for individuals is increasingly attracting psychological attention. However, PTG also attracts controversy as a result of ambiguous empirical findings. The extent that demographic variables, injury factors, subjective beliefs, and psychological health are associated with PTG following ABI is not clear. Consequently, this systematic review and meta-analysis explores the correlates of variables within these four broad areas and PTG. From a total of 744 published studies addressing PTG in people with ABI, eight studies met inclusion criteria for detailed examination. Meta-analysis of these studies indicated that growth was related to employment, longer education, subjective beliefs about change post-injury, relationship status, older age, longer time since injury, and lower levels of depression. Results from homogeneity analyses indicated significant inter-study heterogeneity across variables. There is general support for the idea that people with ABI can experience growth, and that various demographics, injury-related variables, subjective beliefs and psychological health are related to growth. The contribution of social integration and the forming of new identities post-ABI to the experience of PTG is explored. These meta-analytic findings are however constrained by methodological limitations prevalent in the literature. Clinical and research implications are discussed with specific reference to community and collective factors that enable PTG.

## Introduction

Acquired brain injury (ABI) typically occurs as a result of road traffic accidents, assaults or falls, problems in the supply of blood in the brain such as a bleed (hemorrhage) or blockage (stroke), problems in the supply of oxygen (hypoxia) inflammation or swelling of the brain (encephalitis), tumor (meningioma), or surgical issues such as might be involved in tumor management. The incidence of ABI is estimated to be one per 500 of the population globally; children under four, adults under 30, and those over 65 years of age are disproportionately affected (Jones et al., 2011). In fact, ABI is considered one of the most common neurological disorders (Howes et al., 2005). Traumatic brain injury (TBI) is thought to be eight times more common than a combination of breast cancer, AIDS, spinal cord injury, and multiple sclerosis in the USA (Kolb and Whishaw, 2009). Life following ABI is often associated with intense changes including significant social, cognitive, and physical challenges. In addition, people can experience intense changes in identity (Gracey and Ownsworth, 2012). Over 40% of people hospitalized with non-fatal ABI sustain impairments that lead to long-term disability and require acute and post-acute neurorehabilitation to facilitate their appropriate re-adjustment into everyday life (Corrigan et al., 2010). Given the severity of disability and the considerable life expectancy for an ABI survivor, research on ABI and in particular, factors that contribute to the health and well-being of survivors is particularly important. Traditionally, research has focused on the negative consequences of ABI. Whilst this is understandable given that 6 months post-ABI approximately one third of survivors develop clinically relevant psychological distress (Hackett et al., 2005; Bombardier et al., 2010), the fact that a substantial proportion of people with ABI do not develop psychological distress means that positive and protective mechanisms are also worthy of consideration.

Applying positive psychological principles to ABI rehabilitation is growing, encapsulated in a movement that emphasizes “building what's strong” rather than “fixing what's wrong” (Evans, 2011). Over the past 13 years more people are subscribing to the idea that positive growth may be possible after ABI, particularly when changes occur in relation to a person's sense of meaning, purpose, heightened spirituality and enhanced relationships after brain injury (Tedeschi and Calhoun, 2004). In fact, some authors have reported that up to half of their research sample reported post-traumatic growth following the occurrence of ABI (Hawley and Joseph, 2008). Previously, the effects of ABI were seen as irreversible due to a perception that brain injury was a fixed outcome unaffected by the idea of brain plasticity—however, current thinking suggests that social and psychological processes can be harnessed to support and recover brain function to improve outcomes in this population (Walsh et al., 2014). Thus, it is important to seek to understand more about the predictors and processes associated with positive psychological outcomes following ABI. At present, it is unclear what the prevalence of PTG is after injury, what factors predict growth, and what the trajectory of growth might look like for people with ABI.

Until recently there was not sufficient published research on PTG to justify a systematic review. In 2011, Collicutt McGrath published a paper examining the relationship between spirituality and PTG following ABI, including a summary of studies previously published. That article has contributed a solid foundation for us to conduct the first systematic review and meta-analysis of PTG in brain injury survivors, including a more nuanced and thorough analysis of studies published between 1990 and 2014. Here the aim is to address a number of important questions that are previously unanswered in the literature. The review begins by briefly reviewing conceptual and measurement issues in the area of post-traumatic growth. Next, particular attention is paid to the development of PTG in ABI literature and outlining the methods employed here to conduct a systematic review and meta-analysis. The review findings are then presented under four headings. First, the relationship between demographic variables and PTG is considered. Second, the relationship between injury factors and PTG is examined. The remaining two analytic sections consider the relationships between psychological health and PTG, and cognitive processes and PTG. This analysis forms the basis of the subsequent discussion which integrates this work into current conceptual and theoretical debates about PTG and highlights areas where understanding is still poor and/or hampered by methodological controversies. Finally, the implications of this synthesis for clinical practice is considered and an agenda for future research is outlined.

### Current conceptualizations of post-traumatic growth

Positive changes following trauma and adversity have long been recognized in philosophy and religion (Tedeschi and Calhoun, 1995; Tedeschi et al., 1998; Linley and Joseph, 2004), as well as existential (Frankl, 1963; Yalom, 1980) and psychological literature (Park et al., 1996). In research, positive changes have been reported after a range of life challenges (for review, see Linley and Joseph, 2004), including cancer (Collins et al., 1990; Stanton et al., 2006; Cormio et al., 2014, 2015), HIV (Bower et al., 1998), bereavement (Davis et al., 1998), rape (Burt and Katz, 1987; Thompson, 2000), war and conflict (Elder and Clipp, 1989; Waysman et al., 2001), and illness and surgery (Affleck et al., 1987; Tennen et al., 1992). PTG is likely to occur along a continuum, with people differing in their interpretation of the presence and degree of growth experienced. Estimates of perceptions of some degree of growth among people who have experienced psychological trauma typically range from 30 to 80% (Linley and Joseph, 2004).

Three broad areas of positive outcomes after trauma have been identified in the PTG literature. First, individuals report that their relationships with other people are enhanced in some way, including a greater connection to others and greater compassion for others who have suffered. Second, people report changing self-views, including an appreciation of their own personal strength as well as a greater awareness of new possibilities for one's life. Third, individuals report changes in their philosophy about life including changing views about what is important in life. Collectively, these changes have been labeled as post-traumatic growth (PTG: Tedeschi and Calhoun, 1995, 1996), adversarial growth (Linley and Joseph, 2004), benefit finding (Affleck and Tennen, 1996; Tennen and Affleck, 2002; Kangas et al., 2011), and stress-related growth (Park et al., 1996). These terms—particularly PTG and benefit finding—are sometimes used interchangeably. While there are similarities between these constructs, benefit finding is typically described in terms of the acquisition of benefit from adversity, whereas post-traumatic growth is described as the success with which individuals cope or strengthen their perceptions of self, others and the meaning of events after a traumatic event (Brand et al., 2014). Previous studies have also shown that the determinants of PTG and benefit finding are different in other chronic conditions, such as cancer (e.g., Jansen et al., 2011). Thus, for the purposes of the current article, PTG was chosen as the most empirically coherent construct on which to base the meta-analysis.

There has also been confusion regarding the differences between PTG and qualities such as resilience, optimism, hardiness—terms which refer to a person who has adjusted successfully despite adversity (O'Leary and Ickovics, 1995). PTG differs from resilience and recovery in the sense that it is usually understood to refer to an individual moving *beyond* their baseline functioning in terms of relationships, self-views and opening up of life possibilities, rather than simply returning to baseline (Collicutt McGrath, 2011). Throughout this article the term post-traumatic growth (PTG) is used to refer to perceptions of positive changes following a significant life event or trauma (consistent with Collicutt McGrath, 2011), but other terms are used when referring to literature that has used those same terms.

From a theoretical perspective, PTG has been conceived as an outcome of successful accommodation to a traumatic event (Tedeschi and Calhoun, 1995, 2004) and also, as a means of coping with trauma (Taylor and Armor, 1996). Initial conceptualizations of PTG referred to an objective complex cognitive, behavioral and emotional *outcome* after an initial struggle to deal with stressful life circumstances (see Tedeschi and Calhoun, 1995, 2004). Those authors argued that the trauma presents a compelling challenge to basic assumptions about the world, and that PTG occurs when the cognitive schemas that represent these assumptions are rebuilt in a more nuanced and complex form, as a result of the traumatic experience (Collicutt McGrath, 2011). Several models have now been proposed regarding the occurrence and development of PTG. Three comprehensive models exist—Functional Descriptive Model (Tedeschi and Calhoun, 1995, 2004), Organismic Valuing Theory (Joseph and Linley, 2005), and Biopsychosocial-Evolutionary Theory (Christopher, 2004). Although each model has a somewhat different emphasis, each suggests that experiencing a highly stressful or traumatic event shatters an individual's self-views and world-views, and that a meaning-making process or cognitive-affective process occurs in order to adapt or rebuild one's views, resulting in perceptions of growth (Horowitz, 1986; Janoff-Bulman, 2004; Tedeschi and Calhoun, 2004). Most of these theories posit that people are intrinsically motivated toward growth.

Concerns about the theoretical validity of PTG have been raised, where authors suggest that positive cognitive, emotional and behavioral changes are functional illusions. Those researchers (see Taylor, 1983; Affleck and Tennen, 1996; Park and Folkman, 1997; Davis et al., 1998; Filipp, 1999) have argued that perceptions of growth are the result of trying to cope with trauma and reduce feelings of distress. Through this lens, PTG is viewed as a story that we tell ourselves to get throught the challenge, rather than reflecting any real psychological change as a result of struggling with trauma. Other authors (see McMillan and Cook, 2003; Dohrenwend et al., 2004; Cheng et al., 2006) suggest that individuals present themselves in an overly positive light and deny the negative impact of stressful life events, known as defensive denial, as an explanation of PTG. Also, Cognitive Adaptation Theory (Taylor, 1983; Taylor and Brown, 1988) suggests that people have self-protective cognitive biases for seeing positive aspects of negative experiences when they encounter threats—this theory may have relevance for understanding PTG. For example, most people who survive breast cancer report that they are coping as well or better than others facing the same challenge (Wood et al., 1985). Temporal Comparison Theory (Albert, 1977) suggests that individuals make comparisons between their past selves and current selves, and typically distort the past to perceive positive growth. In other words, people sometimes draw the conclusion they are a better version than before (e.g., I am more caring than I used to be). Interpreting PTG as self-enhancing cognitive biases, particularly after ABI where cognitive impairment is often severe, requires a great deal of careful theoretical and empirical attention. It is likely that self-enhancing biases and coping strategies may account for PTG in some individuals. However, It is not possible to distinguish between these processes in the present review.

### Measurement of PTG

Although at least 14 measures of PTG exist, two of the most widely used are the Post-traumatic Growth Inventory (PTGI; Tedeschi and Calhoun, 1996) and the Changes in Outlook Questionnaire (CiOQ; Joseph et al., 1993). The PTGI contains five domains of PTG: (1) new possibilities, (2) relating to others, (3) personal, (4) appreciation of life, and (5) spiritual change. Confirmatory factor analysis has provided further empirical support for this five-factor model (Taku et al., 2008). The CiOQ measures positive changes in the aftermath of trauma in domains similar to that of the PTGI, and has also demonstrated satisfactory psychometric properties (Joseph et al., 2005). There has been some debate in the literature regarding the measurement of PTG. Many scales have been developed to measure growth in response to the incongruencies in its conceptual and theoretical foundations. While overlap exists across these measures, it has been argued that not all are strictly measuring PTG (see Davis et al., 1998; Phipps et al., 2007). The lack of one single definition of PTG has led to measurement difficulties and has caused confusion regarding its correlates, predictors and relation to outcomes. Thus, the current synthesis of existing empirical data on the topic of PTG in ABI, and analysis of the correlates and pathways to growth is particularly timely.

## Method

### Literature search

A computerized literature search was conducted in EBSCOhost on MEDLINE, PsycINFO, PsycARTICLES, CINAHL, AMED, Cochrane Database of Systematic Reviews, Cochrane Central Register of Controlled Trials, EMBASE, Science Direct, Scopus and Web of Science. To ensure adequate coverage of all PTG relevant papers, searches were conducted using the terms “brain injury,” “head injury,” “brain tumor,” “meningioma,” and “stroke” with “posttraumatic growth,” “post-traumatic growth,” “adversarial growth,” “perceived benefits,” “stress-related growth,” “benefit finding,” “positive growth,” “meaning-making,” “positive adjustment,” “finding meaning,” “positive consequences,” “sense-making,” and “thriving.” In addition, the reference lists of all studies included in the review were examined to identify any further relevant articles, as were the reference lists of any systematic reviews identified through this search strategy.

### Inclusion and exclusion criteria

**Figure 1:**
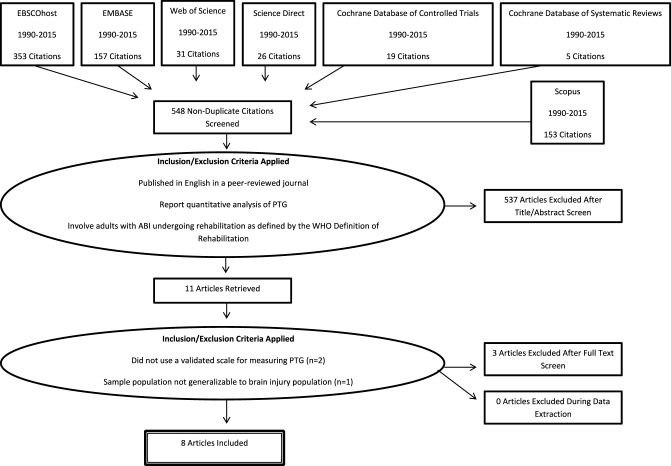
**Number of examined studies and reasons for exclusion by stage**.

To be included in the systematic review, studies were required to meet the following criteria: (1) be published in English in a peer reviewed journal; (2) report quantitative analysis of post-traumatic growth; (3) involve adults with ABI undergoing rehabilitation as defined by the World Health Organization Definition of Rehabilitation; (4) be based within a health-care or community rehabilitation setting. The title and abstract of each article, and the full article where necessary, were independently screened against the inclusion criteria by two reviewers (Jenny J. Grace and Elaine L. Kinsella). In total 744 studies were identified using the search terms, with this number being reduced to eight using the inclusion and exclusion criteria outlined (Figure 1).

The primary reviewers (Jenny J. Grace and Elaine L. Kinsella) independently screened the title and abstract of each article, and the full article where necessary, against the inclusion criteria. Duplicates were removed from the search records (*N* = 196). There was no disagreement among either reviewer as to the final inclusion of studies obtained through the systematic search. Of the 548 studies identified, articles were excluded because participants had not sustained an ABI (*N* = 208), included children or adolescents (*N* = 4), or were review articles, editorials or conference presentations (*N* = 21). Other exclusion criteria included qualitative analysis of the impact of ABI (*N* = 30), articles that did not formally examine PTG (*N* = 211), were animal studies (*N* = 22), erratums and comment articles (*N* = 4), dissertations (*N* = 25), books (*N* = 5) and theoretical articles (*N* = 7). Three further studies were excluded as they did not use a validated scale to measure PTG (*N* = 2), or because the sample was not generalizable to the ABI population (*N* = 1). Of the final eight studies, three contained insufficient information for calculating the effect size (ES^*r*^) for certain constructs. The authors of these articles were contacted, resulting in additional data being provided for one of these studies. In total, the analysis reviewed eight articles with eight independent samples, covering 554 participants and providing 14 ES^*r*^ associations between a variety of constructs and PTG.

### Data extraction

Study characteristics and statistical information were coded into a database by two authors (Jenny J. Grace and Elaine L. Kinsella) using a data coding manual which was developed and revised to include details of the studies (Lipsey and Wilson, 2001). Coded data included methodological factors (sample size, sampling methods, type of measures, study design), sample characteristics (age, time since injury, type of injury), and statistical information for calculating effect sizes (e.g., Pearson correlation coefficient).

From the eight studies included in the analysis, two studies used the same sample at different time points. As recommended by Rosenthal (1995), where samples are not independent significance errors can be avoided by treating the non-independent studies as a single study with several dependent variables. The analysis examines only one dependent variable across studies (PTG) and its association with various independent variables, thus the non-independent samples are treated as *independent*, resulting in eight independent samples.

### Measures

A comprehensive list of measures used to assess PTG and related constructs can be found in Table 1.

**Table 1:** **Demographic and design characteristics of included studies**.

**Study**	***N***	**Design**	**Age**	**Gender**	**Time since injury**	**Measures**
Collicutt McGrath and Linley, 2006	14 Stroke 2 TBI 3 SAH^1^	Cross-sectional	Early group: *M*_age_ = 52 Range = 27–66 Late group: *M*_age_ = 46 Range = 27 – 63	Early group: 4 females, 6 males Late group: 6 females, 5 males	7 months; 10 years	PTGI^2^ SOC-13^3^ HADS^4^
Gangstad et al., 2009	60 stroke	Cross-sectional	*M*_age_ = 71.67, *SD* = 10.64	26 females 34 males	5 – 99 months *(M* = 32.03, *SD* = 23.91)	PTGI CPOTS^5^ HADS
Hawley and Joseph, 2008	165 TBI; 62% severe 15% moderate 23% mild	Longitudinal follow-up 6 months post-recruitment; 10 years	Early group: *M*_age_=32.7, *SD* = 12.98 Late group: *M*_age_ = 34, *SD* = 13.82	Early group: 122 females 441 males Late group: 61 females 104 males	6 months post-recruitment: 2–127 months (*M* = 15.1, *SD* = 22.35) 10 year follow-up: 9–25 years (*M* = 11.5, *SD* = 2.64)	CiOP^6^ Structured interview GOSE^7^ FIM + FAM^8^ HADS COS^9^ ERR^10^
Powell et al., 2007	48 TBI	Cross-sectional	Early group: *M*_age_ = 41.1, *SD* = 13.8 Late group: *M*_age_ = 43.6, *SD* = 13.5	Early group: 4 females 19 males Late group: 5 females 20 males	1–3 years; 10–12 years	PTGI, LSC^11^ HADS BICRO^12^ GOS^7^ Perception of effects of injury
Powell et al., 2012	21 TBI	Longitudinal follow-up; 11 and 13 years post-TBI	*M*_age_ = 42.8, *SD* = 12	2 females 19 males	11 years; 13 years	PTGI, LSC, GOS, HADS, BICRO, PMI^13^, LOT-R^14^, GSES^15^, LOCI^16^, PSS^17^ RBSF^18^, LEQ^19^, OS-CCEI^20^, Perception of effects of injury
Rogan et al., 2013	70 ABI; 56% TBI 31% CVA 13% other	Cross-sectional	Range: 19 – 65, *SD* = 12	21 females 49 males	7–350 months (*M* = 70.43, *SD* = 55.30)	PTGI IPQ-R^21^ Brief COPE HADS FIM + FAM GCS^22^ Demographics
Silva et al., 2011	60 Severe ABI; 58% TBI 42% ABI	Longitudinal follow-up; Discharge and 6 month follow-up	*M*_age_ = 44.18, *SD* = 11.32	16 females 44 males	*M* = 32.92 days (discharge); 6 months	MPAI-4^23^ DASS^24^ PTGI
Zhenxiang et al., 2012	190 Stroke	Cross-sectional	*M*_age_ = 58.57, *SD* = 12.05	72 female 118 male	60% < 6 months 40% > 6 months	PTGI HADS

1 Subarachnoid hemorrhage;

2 Post-traumatic growth Inventory;

3 Sense of Coherence scale-13;

4 Hospital Anxiety and Depression Scale;

5 Cognitive Processing of Trauma Scale;

6 Changes in Outlook Questionnaire;

7 Glasgow Outcome Scale (Extended);

8 Functional Independence Measure and Functional Assessment Measure;

9 Community Outcome Scale;

10 Early referral to rehabilitation;

11 Life Satisfaction Checklist;

12 Brain Injury Rehabilitation Outcome Scales;

13 Personal Meaning Inventory;

14 Life Orientation Test – Revised;

15 Self Efficacy Scale;

16 Locus of Control Inventory;

17 Perceived Social Support;

18 Religious Belief Short Form;

19 Life Event Questionnaire;

20 Obsessionality scale from Crown-Crisp Experimental Index;

21 Illness Perception Questionnaire-Revised;

22 Glasgow Coma Scale;

23 Mayo-Portland Adaptability Scale – 4;

24 Depression Anxiety Stress Scales.

Seven studies utilized the Post-Traumatic Growth Inventory (PTGI; Tedeschi and Calhoun, 1996) to assess PTG, and one study used the Positive Changes in Outlook questionnaire (CiOP; Joseph et al., 1993). The CiOP examines positive psychological change following trauma and adversity and was deemed suitable to include in the analysis as a measure of growth. The variables measured across each of the eight studies were grouped in line with the classifications of demographics, injury and functional variables, psychological health and cognitive processes (see Table 2).

**Table 2:** **ES*r*, confidence intervals, and homogeneity analyses**.

**Variable**	***N***	***k***	**ES_r_**	**95% CI**	***Z* score**	***P*-value for *Z* score**	***Q* statistic**	***P*-value for *Q* statistic**	***I*^2^**
**DEMOGRAPHIC**									
Age	235	2	0.14	0.01,0.26	2.11	0.04	0.05	0.83	0.00
Education	130	2	0.29	0.13,0.44	3.36	0.001	0.18	0.67	0.00
Employment	91	2	0.39	0.20,0.56	3.84	0.00	0.81	0.37	0.00
Gender	235	2	0.01	−0.16,0.18	0.07	0.95	1.55	0.21	35.35
Relationship status	91	2	0.21	−0.001,0.40	1.95	0.05	0.50	0.48	0.00
**INJURY/FUNCTIONAL**									
Activity in community	234	3	0.19	−0.23,0.54	0.87	0.39	13.15	0.001	84.79
Injury severity	283	3	0.01	−0.11,0.13	0.11	0.91	0.81	0.67	0.00
Time since injury	385	6	0.38	−0.04,0.69	1.77	0.08	80.72	0.00	93.81
**COGNITIVE**									
Subjective beliefs	69	2	0.36	0.13,0.55	2.96	0.003	0.14	0.71	0.00
**PSYCHOLOGICAL HEALTH**									
Life Satisfaction	69	2	0.38	−0.27,0.79	1.16	0.24	6.21	0.01	83.90
Anxiety	575	7	−0.07	−0.21,0.07	−1.00	0.32	13.35	0.04	55.05
Depression	635	8	−0.23	−0.37,-0.06	−2.91	0.04	21.99	0.003	68.16

N, total number of study participant; k, number of studies; ES_r_, correlation coefficient effect size; Q, Q statistic which appropriates a chi-square distribution with k − 1 degrees of freedom for test of homogeneity.

### Data synthesis and analysis

#### Effect size calculation

Pearson's product-moment correlation coefficient (*r*) was the primary effect size index used to examine the association between PTG and each of the variables. ES^*r*^ was obtained either directly from the zero-order correlation coefficient reported in the publication, or was extracted and estimated from other reported statistical information (*t*-test, *F* statistic, χ^2^, η^2^, *U*, means, and standard deviations) using DeCoster's effect size calculator (DeCoster, 2012) and Wilson's practical meta-analysis effect size calculator (Wilson, 2001). Where there was insufficient statistical information to calculate an ES^*r*^, authors of the corresponding studies were contacted to obtain this information. If this statistical information could not be obtained, effect sizes were assigned a value of zero and test statistics were reported as *ns.* This represents a commonly used (Helgeson et al., 2006; Sawyer et al., 2010; Quon and McGrath, 2014) but conservative strategy, as effect sizes seldom equal zero. Six associations in the analysis were assigned an effect size of zero. When only a *p*-value was reported, ES^*r*^ was calculated from the *p*-value using an *r*_equivalent_ equation (Rosenthal and Rubin, 2003). If only *p* < 0.05, *p* < 0.01, *p* < 0.001 was reported, an *r*_equivalent_ with *p*-values of 0.0245, 0.005, 0.0005 (one-tailed) was calculated. This method has been shown to provide a conservative estimate of ES^*r*^ (Rosenthal and Rubin, 2003). Where there were two or more ES^*r*^ for one variable within a study, these were averaged to create one ES^*r*^ (Lipsey and Wilson, 2001). Aggregated ES^*r*^ were calculated for the variables of relationship status (Powell et al., 2012) and subjective beliefs about changes post-injury (Powell et al., 2012). An effect size estimation confidence rating (ranging from 1 to 3, with higher numbers representing greater confidence in estimation) was given by the coders to highlight the extent of estimation accuracy of each ES^*r*^ (Lipsey and Wilson, 2001).

#### Analytic strategy

Using Comprehensive Meta-Analysis V2 software program (Borenstein et al., 2005), random effects meta-analytic models were used to examine the association between cognitive processes, psychological health, demographic and injury variables, and PTG. Random effects models assume that each sample is drawn from a population with different effect sizes and thus allows for both random variance and variance due to true differences between the populations. Random effects models also permit generalization of inferences due to this explicit inclusion of between-study variability (Lipsey and Wilson, 2001; Borenstein et al., 2009).

Aggregated ES^*r*^ statistics were calculated for the variables where two or more studies investigated the association between a variable and PTG. The homogeneity of ES^*r*^ in each meta-analytic model was examined using the *Q* statistic (Lipsey and Wilson, 2001) and *I*^2^ statistic (Borenstein et al., 2005). The *Q* statistic measures the variation of ES^*r*^ in each model, where a non-significant *Q* statistic indicates a homogenous distribution (Borenstein et al., 2005). The *I*^2^ statistic estimates the percentage of between-study variability due to heterogeneity rather than chance (Borenstein et al., 2005).

In order to test for the presence of publication bias, Rosenthal's *fail-safe N* (Rosenthal, 1979) was employed. This technique involves estimating the number of unpublished studies reporting null results required to overturn the results of the meta-analysis. A higher number of studies indicate a more robust estimate of ES^*r*^. Publication bias was examined for each variable by funnel plot (Stearne et al., 2005).

## Results

### Study characteristics and quality

The research design of each study is summarized in Table 1. Studies meeting the inclusion criteria were published between 1990 and 2015. Sample sizes ranged from 19 to 190 participants. Two studies examined PTG in individuals who had suffered a stroke, three studies examined PTG in those who sustained a TBI, and three examined PTG in individuals who suffered a stroke, TBI, or “other” ABI causes such as encephalitis or subarachnoid hemorrhage.

Detailed demographic information for the included studies is listed in Table 1. Overall, 39% of participants were female and 61% were male. Participant ages ranged from 16 to 88 years. The mean length of education for participants was reported in only three studies (*M*_years_ = 14.19, *SD* = 1.13).

The quality of each study was determined on the basis of four study characteristics as developed by Ferro and Speechley (a modified version of the quality index; Ferro and Speechley, 2009): (i) reporting, (ii) internal validity, (iii) external validity, and (iv) power. This quality index comprises four subscales consisting of 15 items: reporting (0–7), external validity (0–3), and internal validity (0–4), and power (assessed with a single item, 0–1). For each of the eight studies included in the analysis, each item was scored 0 (no/unable to determine) or 1 (yes). Studies could achieve a maximum score of 15. Higher scores on the quality index indicated greater methodologic quality. Table 3 provides an overview of scores achieved by each study in each of the domains measured by the quality index. The total mean score on the modified quality index was 11.38 (*SD* = 1.86, range 8–14). The mean subscale scores were 5.25 (*SD* = 1.24, range 3–7) for reporting, 2.63 (*SD* = 0.72, range 1–3) for external validity, 3.50 (*SD* = 0.51, range 3–4) for internal validity, and scores of zero for power. The intra-class correlation (ICC = 0.952) indicates very high interrater agreement for study quality between both coders (Jenny J. Grace and Elaine L. Kinsella). Table 4 includes a summary of mean PTG scores across ABI and other trauma-related populations.

**Table 3:** **Study quality assessment (Ferro and Speechley, 2009)**.

	**Reporting (0–7)**	**External validity (0–3)**	**Internal validity (0–4)**	**Power (0–1)**	**Total (0–15)**
Collicutt McGrath and Linley, 2006	4.5	1	3	0	8.5
Gangstad et al., 2009	7	3	4	0	14
Hawley and Joseph, 2008	5.5	3	3	0	11.5
Powell et al., 2007	3	3	3	0	9
Powell et al., 2012	5	3	3	0	11
Rogan et al., 2013	6	3	4	0	13
Silva et al., 2011	5	3	4	0	12
Zhenxiang et al., 2012	6	2	4	0	12
Total	42	21	28	0	91

**Table 4:** **Summary of mean PTG scores for ABI and other events**.

**Study**	**Event**	**N**	**Measure**	**Mean (*SD*)**
**ABI STUDIES**				
Collicutt McGrath and Linley, 2006	Stroke TBI^*^ SAH^**^	Early: 4 female, 6 male Late: 6 female, 5 male	PTGI	Not reported Not reported
Gangstad et al., 2009	Stroke	26 female 34 male	PTGI	50.33 (19.92)
Hawley and Joseph, 2008	TBI^*^	Early: 122 female 441 male Late: 61 female 104 male	CiOP	43.41 (10.76)
Powell et al., 2007	TBI^*^	Early: 4 female 19 male Late: 5 female 20 male	PTGI	36.50 (18.70) 68.1 (16.60)
Powell et al., 2012	TBI^*^	2 female 19 male	PTGI	64.6 (16.50)
Rogan et al., 2013	TBI^*^ CVA^***^ Other	21 female 49 male	PTGI	53.76 (22.88)
Silva et al., 2011	TBI^*^ ABI^****^	16 female 44 male	PTGI	33.47 (18.26)
Zhenxiang et al., 2012	Stroke	72 female 118 male	PTGI	58.10 (13.72)
**OTHER EVENTS**				
Calhoun et al., 2000	Various	54	PTGI	76.5 (22.00)
Cordova et al., 2001	Breast cancer	70 Female 70 Healthy comparison (Female)	PTGI	64.1 (24.80) 56.3 (26.30)
Polatinsky and Esprey, 2000	Bereaved of child	49 Female 18 Male	PTGI	83.47 (20.21) 79.72 (19.50)
Snape, 1997	Accident/ assault	13 Female 40 Male	PTGI	52.15 (25.59) 55.43 (18.14)
Tedeschi and Calhoun, 1996	Various	405 Female 199 Male	PTGI	75.18 (21.24) 67.77 (22.07)
Tedeschi and Calhoun, 1996	Various	62 Female 55 Male	PTGI	81.60 (21.09) 70.25 (21.87)
Weiss, 2002	Breast cancer	41 Female 41 Male	PTGI	60.21 (18.81) 46.00 (22.83)

* Traumatic brain injury;

** Subarachnoid hemorrhage;

*** Cerebrovascular accident;

**** Acquired brain injury.

### Mean effect sizes

A stem-and-leaf plot of all effect sizes in the analysis is presented in Table 5.

**Table 5:** **Stem-and-Leaf plot of all effects sizes (*r*)**.

**Stem**	**Leaf**
−0.5	3
−0.4	2
−0.3	1
−0.2	0,9
−0.1	8
−0.0	1,2,3,3,4,6,6,9
0.0	0,0,0,0,0,2,8,9,9
0.1	0,0,2,3,6,7
0.2	6
0.3	3,5,5,6
0.4	2,6
0.5	3,4
0.6	5,7
0.7	
0.8	
0.9	7

For demographic variables, ES^*r*^ ranged from 0.01 to 0.39, for injury and functional variables ES^*r*^ ranged from 0.01 to 0.38. Cognitive processes demonstrated ES^*r*^ of 0.36 and psychological health variables demonstrated ES^*r*^ ranging between −0.23 and +0.38 (see Table 2).

### Publication bias and tests of homogeneity

Rosenthal's (1979) *fail-safe N* was used to estimate the effect of publication bias in the analysis. Begg and Mazumdar's (1994) rank correlation test is usually preferred for this purpose but can lack power for smaller meta-analyses (Rothstein et al., 2005). Publication bias could not be estimated for age, life satisfaction, employment, education, subjective beliefs about changes post-injury, relationship status and gender due to the limited number of studies examining the relationship between each of these variables and PTG. For depression, Rosenthal's *fail-safe N* was 55, and for time since injury *fail-safe N* was 34 (Table 6).

**Table 6:** **Rosenthal's *fail-safe N* for estimation of publication bias**.

**Variable**	**Number of observed studies**	**Fail-safe N**
Activity in community	3	0
Anxiety	6	0
Depression	8	55
Injury severity	3	0
Time since injury	6	34

Given the original number of observed studies for each of these variables, this would indicate that the ES^*r*^ for these variables are reliable estimations. Rosenthal's *fail-safe N* was 0 for each of the following variables: activity in community, anxiety, and injury severity. These results would indicate that publication bias likely impacted the ES^*r*^ of these variables and as a result, the robustness of these ES^*r*^ estimations.

Results from homogeneity analyses indicated significant inter-study heterogeneity for the variables of: activity in the community, time since injury, life satisfaction, anxiety and depression. The magnitude of the observed *Q* statistics indicated variable levels of unexplained heterogeneity across ES^*r*^, and the *I*^2^ statistic for these variables demonstrated the percentage of inter-study variability due to heterogeneity rather than chance. The remainder of the variables demonstrated non-significant *Q* statistics, suggesting that the ES^*r*^ for these variables were homogenous (see Table 2). In the presence of unexplained heterogeneity, further exploration of potential moderators may be suggested (Rosenthal, 1995). However, due to the relatively small number of studies included in each of the current analyses, such moderator analyses were beyond the scope of this review (Tabachnick and Fidell, 2007).

Results from the meta-analysis indicated small to medium effect sizes across all examined variables. Positive associations were evident for demographic variables and cognitive processes, with both positive and negative associations demonstrated for psychological health and injury variables (see Table 2). In particular, results demonstrated that subjective beliefs about change in one's life post-injury, longer duration of education, being employed, older age, longer time since injury, being in a relationship, and lower levels of depression are significantly associated with PTG after ABI.

## Discussion

In the next section, these meta-analytic findings relative to four key dimensions are discussed: demographic factors, injury level variables, psychological health and cognitive processes.

### Demographic factors and PTG

#### Age of participants

Age demonstrated a small effect size indicating that older individuals report greater levels of PTG than younger persons (the average age of participants in the current analysis was 46 years). A curvilinear relationship between age and PTG following ABI may exist (see Thompson, 1991), where persons in the mid-stage of their life are best placed to abstract positive change from the experience of their ABI. In contrast, previous meta-analyses have reported a general trend, in non-ABI samples, toward younger persons experiencing greater levels of PTG following trauma or illness than older adults (Helgeson et al., 2006; Barskova and Oesterreich, 2009; Sawyer et al., 2010). For this reason, some authors posit that being diagnosed with a serious medical condition or experiencing trauma at a younger age may implode one's worldview of natural and fair social order, thus allowing for a greater reconstruction of previously held worldviews and act as a catalyst for growth (Helgeson et al., 2006; Sawyer et al., 2010). However, some inconsistencies in the relationship between age and PTG have been acknowledged in the literature (Barskova and Oesterreich, 2009). Those authors suggest that age may influence the processes by which growth occurs at the onset of an illness or life-altering event, and suggest that different questions on the PTGI scale may be more applicable and relevant at different life stages. For instance, younger people may report greater levels of “new possibilities,” whereas older adults may relate to other items more strongly.

#### Employment

Employment demonstrated a medium effect size in the analysis indicating that employment is associated with greater levels of growth. The ABI literature has reported that employment is associated with greater perceived well-being, improved social integration within the community, more frequent pursuit of leisure and home activities, and greater health status, less usage of health services, more social contact, greater autonomy and a clearer sense of personal identity (Webb et al., 1995; O'Neill et al., 1998, 2004; Corrigan et al., 2001; Steadman-Pare et al., 2001; Wehman et al., 2005). The World Health Organization's International Classification of Functioning, Disability and Health (IFC) highlights that returning to work is a key component of rehabilitation and should not remain a marginal outcome of recovery (WHO, 2001). Powell et al. (2012) reported that participants who demonstrated higher levels of PTG more frequently reported that they were able to work. Employment following ABI may help to develop social support systems that provide a buffer against distress and allow a person to derive meaning from their ABI, thus increasing perceptions of PTG—however, these processes remain underexplored.

#### Education

The analysis demonstrated a medium effect size for education such that people who reported a longer duration of pre-injury education experienced greater levels of PTG. Barskova and Oesterreich (2009) reported that level of education was unrelated to PTG in a sample of people with serious medical conditions, but highlighted limitations in the sample distribution for education in their review. Gangstad et al. (2009) demonstrated that education predicted PTG in persons who had sustained an ABI. Theories of cognitive reserve (Satz, 1993; Stern, 2003) have attempted to explain why, in the face of similar objective injury severity and injury location, the impact of ABI on cognitive ability differs across individuals. These theories suggest that education may act as an aspect of reserve that maintains greater levels of cognitive functioning despite objective injury severity.

#### Gender

There was a very small effect size for gender in the analysis. Recent meta-analyses demonstrated greater levels of PTG in women than in men (Barskova and Oesterreich, 2009; Vishnevsky et al., 2010), however these results appeared to depend on the type of measure used to examine PTG (Barskova and Oesterreich, 2009). Two reviews (Helgeson et al., 2006; Sawyer et al., 2010) found that gender did not moderate the relationship between PTG and positive psychological adjustment, depression, intrusive-avoidant thoughts, and subjective physical health, and reported that significant variability remained present when gender was examined as a moderator in these relationships.

#### Relationship status

Results indicated a small effect size for relationship status and PTG. Similarly, Helgeson et al. (2006) reported a very small effect size for the association between marital status and PTG following health-related or personal trauma. Updegraff and Taylor (2000) propose that marital status should be associated with positive psychological growth following loss or trauma through the support system provided by a close relationship. Indeed, a review of the literature relating to stress and coping among families following TBI demonstrated a positive link between family member coping and recovery for the person with TBI (Verhaeghe et al., 2005). On a different but related topic, results from Powell et al. (2012) demonstrated that being in a new relationship since sustaining an ABI differentiated between persons high and low in PTG, while being in the same relationship as before injury did not. Perhaps being in a new relationship facilitates a person with ABI to develop a new positive identity after injury and boost perceptions of PTG, while also reducing the likelihood of temporal comparisons between pre- and post-injury selves. Alternatively, it is possible that a person who reports PTG is more open to forming new social bonds and interpersonal relationships than those who have not experienced growth. Interestingly, Ackroyd et al. (2011) reported that PTG in persons with multiple sclerosis tended to be predicted by PTG in their partners, reiterating the importance of constructive social relationships on positive psychological outcomes. These findings are complex but suggest a fruitful avenue of future research investigating the relationship between social capital and PTG following ABI.

### Characteristics of ABI and PTG

#### Time since injury

Results demonstrated a medium effect size for time since injury and PTG highlighting that over time people with ABI experience more growth. In the current analysis, the average time since injury was 5.6 years. Findings across reviews of the temporal course of PTG in diverse samples are inconsistent and seem to be a function of methodological differences across studies—for instance, the use of a cross-sectional or longitudinal research design, the measures used, the type of trauma in question, and the different time points when participants were assessed after the trauma or challenging life event. Helgeson et al. (2006) and Sawyer et al. (2010) found that time since trauma was a significant moderator in the relationship between PTG and both positive and negative mental health. Specifically, Sawyer et al. (2010) found that in the early years following trauma, PTG appeared to play a role in reducing the negative effects of trauma but as time passed, PTG appeared to enhance well-being. Research has demonstrated that across an entire sample of participants who were between 7 months and 10 years post-ABI, an enhanced appreciation for life was the most endorsed aspect of PTG, followed by relating to others, the realization of personal strengths, new possibilities, and spiritual change (Collicutt McGrath and Linley, 2006).

#### Injury severity

Injury severity demonstrated a small effect size for PTG. Importantly, the literature on PTG suggests that it is the subjective appraisal of a threatening event rather than its objective characteristics that are associated with growth (Tedeschi and Calhoun, 1995, 2004; Linley and Joseph, 2004). In the context of ABI, one might expect that a severe injury is likely to affect a person's ability to engage in the cognitive processes theorized to be required for growth. Powell et al. (2012) reported that having a mild level of disability as a result of ABI differentiated between high and low levels of PTG. Interestingly, most of the overall sample in the current analysis sustained severe brain injuries but reported levels of growth comparable to and greater than those with less severe head injuries (Powell et al., 2007; Hawley and Joseph, 2008; Rogan et al., 2013). This finding is consistent with arguments that high levels of trauma and distress are needed to provoke perceptions of PTG (see Cognitive Processes section below for further discussion).

#### Activity in the community

The analysis demonstrated a small effect size for activity in the community and PTG. Activity in the community was measured across studies using validated scales that examined a person's engagement in paid and voluntary work, study or looking after children, and in terms of mobility, occupation, engagement, and social integration. This is an important aspect of brain injury rehabilitation as people often report isolation and reduced social support following ABI (Johnson and Davis, 1998).

Research on the relation between activity in the community and PTG is relatively sparse. Chun and Lee (2008) qualitatively identified that the experience of meaningful engagement in activities was one of the most salient themes of PTG in a sample of individuals with spinal cord injury. Further, they identified that meaningful engagement involved the recognition of personal strengths, experience of strengthened social relationships through activities, and experience of positive emotion. Activity in the community following ABI may represent a form of meaningful engagement by giving a person a sense of purpose and social identity through work-based activities (Haslam et al., 2000), and may facilitate the creation of new social networks and support systems which in turn promote growth.

### Psychological health

#### Life satisfaction

The meta-analysis demonstrated a medium effect size for the relationship between life satisfaction and PTG. Findings across the literature have been inconsistent regarding the relationship between well-being and PTG (Zoellner and Maercker, 2006). Recent research has demonstrated that life satisfaction is indirectly related to levels of PTG through the sense of meaning and purpose that growth can imbue following a traumatic event (Triplett et al., 2012).

Results of our analysis reveal that following ABI people who report growth also report life satisfaction and psychological well-being. Meta-analyses examining psychological well-being and PTG in cancer or HIV/AIDS (Sawyer et al., 2010) and personal or other health-related trauma (Helgeson et al., 2006) have reported positive associations between these two variables. One meta-analysis revealed that when time since trauma was greater than 2 years, PTG was more strongly related to positive well-being (Helgeson et al., 2006). A strong relation between life satisfaction and PTG was demonstrated 11–13 years post-TBI (Powell et al., 2012), while another study demonstrated that 1–3 years and 10–12 years post-TBI there was no such association (Powell et al., 2007). This finding is instrumental in the context of ABI, as outcomes following brain injury can be poor (Langlois et al., 2006; Bazarian et al., 2009). The opportunity for psychological well-being following trauma adds a new dimension to brain injury rehabilitation and sentiments of “building what's strong” rather than “fixing what's wrong” (Evans, 2011).

#### Depression

Our analysis revealed a small effect size for the relationship between depression and growth. To date, research has not demonstrated a consistent relationship between depression and PTG cross-sectionally, with mean correlation coefficients ranging between −0.1 and 0.1 (Linley and Joseph, 2004; Zoellner and Maercker, 2006). Barskova and Oesterreich (2009) reported that eight (out of 15) cross-sectional studies and four longitudinal studies examining the association between depression and PTG in individuals with serious medical conditions found a negative relationship between depression and growth.

In Tedeschi and Calhoun's (1995, 2004) model of PTG, it is assumed that the initial distress associated with a traumatic event is fundamental in the process of catapulting the individual in a search for meaning, which initiates cognitive processing that is used to make sense of the trauma and its related consequences. They suggest that this initial distress maintains cognitive processing, and the sometimes lengthy period during which distress persists may be fundamental to the occurrence of maximum levels of growth. Helgeson et al. (2006) and Sawyer et al. (2010) reported that the relationship between depression and PTG was moderated by time since event. Specifically, Helgeson et al. (2006) reported that 2 years or less post-trauma, PTG was related to more global distress, however lower levels of depression and greater positive affect were correlated with greater levels of PTG when time since event was more than 2 years. Gangstad et al. (2009) reported a positive relationship between depression and anxiety and PTG in the early stages following a stroke, which became more significant and negative over time. It is likely that a person with ABI may face ongoing difficulties and traumatic periods while they adjust to the physical, psychological, and social changes occurring in their lives (e.g., further cognitive impairment as a result of a seizure or a relationship breakdown many years after the onset of injury). The extent that existing research methodologies and measures of PTG capture the often ongoing set of challenges presented by ABI is still unclear.

#### Anxiety

The present analysis demonstrated a very small effect size for the relationship between anxiety and PTG. The wider literature has mainly demonstrated no relationship between anxiety and growth (Helgeson et al., 2006), however this has varied depending on the type of trauma experienced by the individual, with some studies reporting a positive relationship between anxiety and PTG (Barskova and Oesterreich, 2009). Given that the present analysis demonstrated a very small effect size for anxiety and PTG and the inconsistent findings in the wider literature, anxiety may not play a prominent role in the development of PTG in ABI, but may be part of a wider set of interrelations that promote growth.

### Cognitive processes

Theories of PTG, whether conceptualized as a coping process or the outcome of a struggle with adversity (Tedeschi and Calhoun, 1995, 2004; Affleck and Tennen, 1996) suggest that the concept of growth is underpinned by subjective appraisals of a traumatic event. The relationship between cognitive processes and PTG in the wider literature is complex. In an examination of the presence of a two-component model of PTG (the “Janus-face” model of PTG) as a potential explanation for the often inconsistent results reported in the empirical PTG literature, Maercker and Zoellner (2004) and Zoellner and Maercker (2006) suggest that different cognitive processes (constructive vs. illusory) may be involved at different times in the growth process. Furthermore, different cognitive processes may relate differently to PTG and outcomes following trauma. Given that people who have sustained a moderate to severe brain injury are highly likely to experience cognitive impairment (Cicerone et al., 2011), this population may be very well placed to permit examination of the extent to which growth can be experienced, particularly in light of theories of PTG that highlight the importance of cognitive processes in the development of growth (Tedeschi and Calhoun, 1995, 2004; Linley and Joseph, 2004).

#### Subjective beliefs about changes post-injury

The present analysis demonstrated a medium effect size for the relationship between subjective beliefs about changes post-injury and PTG following ABI. Two studies (Powell et al., 2007, 2012) have considered subjective beliefs about changes post-injury, under the heading “perception of effects.” This variable concerned the extent to which participants agreed with two polarized statements: “the effects of my head injury have meant that in some ways my life has been richer and fuller” and “the effects of my head injury have ruined my life.” Powell et al. (2007) found that greater agreement with the statement “the effects of my head injury have meant that in some ways my life has been richer and fuller” was significantly positively correlated with PTG, but did not find a significant correlation between “the effects of my head injury have ruined my life” and PTG. Similarly, Powell et al. (2012) reported that positive subjective beliefs about changes post-injury (i.e., perception of effects) were significantly correlated with PTG. As such, it would seem that how a person perceives the effects of their ABI is crucial to growth.

#### Subjective beliefs about changes post-injury, severity of injury and PTG

Powell et al. (2007) reported that individuals who fell into the “severe” category of severity of injury and disability agreed significantly more with the statement “the effects of my head injury have ruined my life” compared to those who were classed as having a “moderate” or “mild” level of injury severity and disability. It would seem obvious to suggest that a severe brain injury would confer greater levels of disability and life changes and thus, the effects of these changes might amount to the interpretation of one's life being ruined. Yet, there were no significant differences across levels of severity and disability and the perception that one's life has been richer and fuller as a result of brain injury. Further evidence for the importance of subjective beliefs about changes post-injury in the development of PTG can be found in Powell et al. (2012), where subjective beliefs of one's life as richer and fuller differentiated between those who reported high and low levels of PTG. Silva et al. (2011) also reported that subjective impairment at discharge following ABI was positively associated with PTG at 6-months follow-up.

#### Illusory mechanisms and PTG

The idea that growth may not reflect genuine changes in terms of meaning, new life priorities, relationships or an enhanced appreciation for life but instead may represent a self-preservation or illusory coping strategy has gained momentum in light of the often contradictory and inconsistent relationships evident in the empirical study of PTG (see Taylor and Brown, 1988; Taylor et al., 2000; Zoellner and Maercker, 2006; Sumalla et al., 2009). While many of the studies in the current analysis reported greater levels of PTG over time, only Gangstad et al. (2009) examined potentially illusory cognitive mechanisms that may serve to preserve a person's self- and world-views in the initial stages following brain injury. In line with literature examining the potential for co-existing adaptive and maladaptive types of growth (Zoellner and Maercker, 2006; Sumalla et al., 2009; Sawyer et al., 2010), Gangstad et al. (2009) reported that denial and downward comparison—a process in which an individual compares their situation to that of another who they perceive to be less fortunate as a way to draw value from their own situation—were both associated with reports of PTG; denial was shown to predict growth. The mean time since ABI for the sample in the study was 32 months and as such could be regarded as early in the process of recovery. At this stage, people may use denial as a coping strategy to reduce the levels of distress they are experiencing as a result of their injury. Interestingly, they also observed that as time since injury increased levels of depression reduced, while engagement in downward comparison increased. Perhaps in this instance, PTG may represent a “palliative coping strategy” (see Zoellner and Maercker, 2006) where the proposed illusory mechanism at play is paving the way for future genuine growth by allowing the person to perceive positive aspects by comparing themselves to those they perceive as less fortunate than themselves. This may promote voluntary use of adaptive coping strategies over time, leading to a reduction of distress in the short-term, and an increase in genuine levels of growth over time.

The goal of the current article was to examine the correlates of PTG following brain injury and to assess the value of future examination of the interplay between the complexities of ABI and PTG. Overall, the current analysis revealed that less depression, relationship status, employment, longer duration of education, longer time since injury, subjective beliefs about change post-injury, and older age are significantly associated with PTG following ABI.

### Limitations

The results of this meta-analytic review should be interpreted with the following limitations in mind. While the analysis provided us with average effect sizes across included studies, the relatively small number of empirically acceptable studies published on the topic of PTG in brain injury left us with a limited number of studies to include in the analysis and as such, it was not possible to examine moderator variables in the analysis.

It is important to bear in mind that both cross-sectional and longitudinal studies were included in this meta-analysis. The wider growth literature has highlighted that cross-sectional and longitudinal research designs may yield different results across relationships with PTG (Zoellner and Maercker, 2006; Sumalla et al., 2009). While the inclusion of longitudinal studies in the analysis provides some evidence for the increase of PTG over time, the interaction with results from cross-sectional studies is unknown. Moderator analysis that investigates study design and study quality as moderators in the relationships between variables may have shed more light on effect of methodological practices on results.

Where authors did not report sufficient statistical information to calculate effect sizes and this information could not be obtained from researchers directly, the effect sizes were coded as zero. This is a very conservative approach and as such may have impacted the effect size for the analyses that included these “zero” effect sizes. Second, only published studies were included in the analysis. Where the analysis allowed us to conduct publication bias analyses, Rosenthal's *fail-safe N* highlighted significant publication bias for a number of effect sizes. There may be methodologically strong but unpublished studies in this area which if included in the analysis may have had an effect on the results of the current analysis.

The term PTG was originally applied to survivors of war and natural disasters, and other one-off traumatic events. The extent that the PTGI accurately captures the often ongoing and non-linear levels of distress after brain injury is not confirmed. For example, a survivor of ABI may continue to have seizures many years after the first diagnosis of ABI which may cause further physical and cognitive decline, and in turn the survivor's perceived levels of PTG may appear to fluctuate. Also, recent reports in the literature indicate that cultural factors influence the development of post-traumatic growth (Shakespeare-Finch and Copping, 2006; Cormio et al., 2014). The studies included in this analysis involve participants sampled in the USA, Ireland, China, and the UK, but unfortunately detailed cross-cultural comparisons are beyond the scope of the available data. There is a growing need to pay attention to the patterns of growth after brain injury across cultures and the extent that PTGI is a suitable measure of growth in non-USA samples.

### Clinical implications

It is expected (but not assumed) that many people will experience their ABI as a traumatic event. PTG offers us an alternative way to view trauma (Joseph, 2012), and the evidence suggests that many people with ABI *do* report PTG. The possibility that growth may be experienced by persons with ABI is a far departure from a traditional approach of focusing on disability and deficit during neurorehabilitation. Recognition that traumatic events may in time engender growth may permit an additional consideration of the manner in which wider systemic, structural and contextual factors impact on positive appraisal processes underpinning PTG in people with ABI. As such, clinicians, health professionals and carers could look for potential ways to instigate and facilitate positive and meaningful changes in the lives of people with ABI (Linley and Joseph, 2004; Tedeschi and Calhoun, 2004). For instance, clinicians could promote the use of adaptive cognitive processing strategies, including deliberate rumination and positive cognitive re-appraisal to engender a new perspective. Gaining a new perspective of a changed reality may facilitate the use of adaptive coping strategies and the instigation of PTG following ABI (Rogan et al., 2013).

Furthermore, laying out realistic prospects of a person's post-injury recovery trajectory, which may include the possibility of growth, could help to manage the individual's expectations of a potentially non-linear journey including both distress and growth. Specific strategies that support the communal search for meaning (Ackroyd et al., 2011) where the person with ABI and their partner attempt to make sense of the significant changes together may be an additional powerful instigator of change for persons in committed relationships.. In addition, active plans to support persons with ABI in their return to productive roles including work, training or other meaningful occupations may be instrumental in helping the individual to build a new social identity, social network, and provide a platform for PTG.

### Future directions

How do individuals who have sustained cognitive deficits as a result of ABI experience growth, when the basis for growth is proposed to rely on cognitive processing of a traumatic event? Severity of injury demonstrated a very small effect size for PTG in the analysis. Most of the sample in the analysis sustained severe brain injuries yet reported levels of growth similar to those who experienced different types of trauma (see Table 4). Future research could examine the interaction of specific cognitive difficulties, or indeed particular brain lesion locations, and reports of PTG. In addition, many studies did not measure participants' levels of self-awareness. Future research could aim to examine the relationship between self-awareness, specific cognitive difficulties, and PTG.

Clinical interventions for rebuilding identity following ABI demonstrate meaning-making dimensions (e.g., meaning centered therapy, Gracey et al., 2008; client-focused and value-driven approaches, Muenchberger et al., 2008) and are placed within the social context. PTG involves a significant meaning-making aspect, where an attempt to make sense of one's circumstances may lead to growth (Tedeschi and Calhoun, 1995, 2004) and well-being (Triplett et al., 2012). An interesting area of future research could examine the relation between identity and PTG following ABI, the social context in which this occurs, and how social factors interact with identity development and PTG after ABI.

## Conclusions

The current study has extended the literature by highlighting correlates of PTG in the context of ABI. The analysis demonstrated that while significant inter-study heterogeneity across variables was extant, subjective beliefs about changes post-injury, greater levels of education and employment, older age, relationship status, time since injury, and lower levels of depression are related to PTG following ABI. The findings from this meta-analytic review have important implications for rehabilitation planning, and in particular highlight that ABI not only represents negative life changes, but can also demonstrate an “existential heart to trauma” (Frankl, 1963). A great deal of future research is needed to examine the extent that persons perceive their brain injury as traumatic, the extent that growth is perceived following ABI, and the consistency and causality of relationships between PTG and other variables. An interesting and perhaps previously overlooked notion is that social relationships, as well as the creation of a positive social identity after ABI, may play a role in perceptions of growth and positive adjustment. Such social capital aspects of experience are likely to enhance attainment of more distal rehabilitation goals such as improved community integration and participation for people with ABI (Larsson et al., 2013). Indeed given that the final endpoint of rehabilitation is the person's integration and participation in their social community, the social and communal aspects of growth reviewed in this paper may hopefully add to the evidence base for promotion of PTG as an increasingly legitimate focus for post-acute rehabilitation. Further exploration of the nature and predictors of PTG and other meaning-based coping efforts that might improve the quality of research evidence and ultimately result in improved outcomes for people who are living with ABI is encouraged.

### Conflict of interest statement

The authors declare that the research was conducted in the absence of any commercial or financial relationships that could be construed as a potential conflict of interest.
